# Vertebral body chondrosarcoma with metastasis to the scalp

**DOI:** 10.1259/bjrcr.20180037

**Published:** 2018-07-12

**Authors:** Aya Fukuda, Dayvid L de Castro Oliveira, Andrei Fernandes Joaquim, Eliane Maria Ingrid Amstalden, Luciano de Souza Queiroz, Fabiano Reis

**Affiliations:** 1 Master Student in Oncology, State University of Campinas, Campinas, Brazil; 2 Department of Neurosurgery, State Universityof Campinas, Campinas, Brazil; 3 Department of Pathology, State University of Campinas, Campinas, Brazil; 4 Department of Radiology, State University of Campinas, Campinas, Brazil

## Abstract

We present a case of a 30-year-old man who had a 3-year history of low back pain. MRI demonstrated an infiltrative mass, affecting the vertebral body and pedicles of L4, with some extension to the vertebral canal. There was also tumor invasion in the inferior vena cava and in the left iliopsoas muscle. The anatomopathological examination of the resected L4 vertebral body was of a malignant neoplasia compatible with mesenchymal chondrosarcoma (high histological grade). About 2 months after surgery, he developed a progressive bladder incontinence, bilateral leg weakness and severe back pain. A new MRI was obtained, confirming progression of the disease. An occipital scalp lesion was detected and biopsy confirmed cutaneous metastasis. Primary malignant bone tumors are rare but should be ruled out in young patients with persistent low back pain. We present a case of a confirmed mesenchymal chondrosarcoma affecting lumbar spine, with MRI and pathological illustrations. Early diagnosis may improve the chances of local disease control and even cure.

## Introduction

Metastases are the most frequent bone tumors (reaching about 25% of cases).^1^ Spinal column is the most common bone affected by metastases of many primary malignant tumors, corresponding to the third most frequent site after liver and lung.^1^ Primary bone tumors of the spine are rare (about 5% of all bone tumors).^2^ The most common malignant primary bone tumors are multiple myeloma and other lymphoproliferative tumors.^3^ Among the non-lymphoproliferative primary malignant tumors that affect the spine, the most common is vertebral chordoma, followed by chondrosarcoma.^4^ Primary chondrosarcomas of the vertebral column are rare, generally located in the posterior vertebral elements (in about 40% of cases). Vertebral bodies are affected in 15% of cases.^5^

A high level of suspicion is necessary since these tumors are rare, especially in young patients with low back pain. Symptoms of secondary low back pain should be considered, such as pain at night, loss of weight, refractory pain, neurological involvement. MRI should be considered for investigation.^4^ Here, we present a case of a young patient with a high-grade chondrosarcoma of the mesenchymal type.

## Case report

A 30-year-old male patient was examined in our outpatient clinic with a 3-year history of mild low back pain. In the last 20 days, the pain worsened severely, with irradiation to the posterolateral aspect of the lower limbs and difficulty to walk. Palpation of the lumbar spine sacroiliac joints was painful (positive Patrick’s test). Laboratory analysis showed normal levels of inflammatory markers (normal reactive C protein).

**Figure 1.**
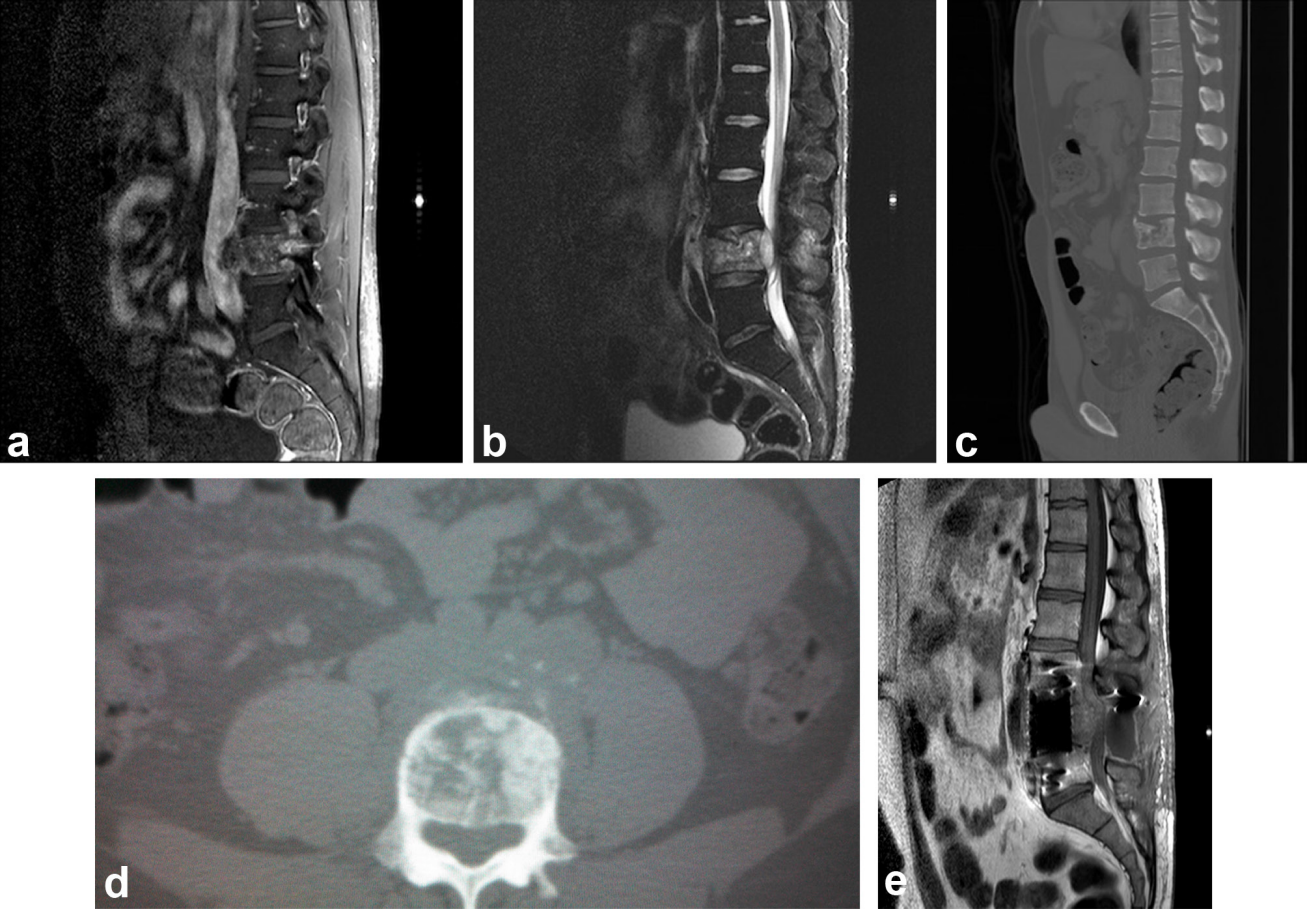
MRI and CT of mesenchymal chondrosarcoma in vertebral body. (a) Sagittal *T*_1_ fat-sat after contrast revealed a vertebral body lesion with heterogeneous contrast enhancement, extending to the L4 pedicle. (b) Sagittal STIR image showing extension of the lesion at the level of L4, with epidural component that occupies the vertebral canal, displacing the *cauda equina* roots and determining spinal stenosis. (c) Sagittal CT shows a lytic lesion with indistinct borders, located in the vertebral body of L4, associated with a soft tissue component that displaces the aorta and the vena cava and infiltrates the vertebral canal. (d) CT in the axial section shows typical “ring-and-arc” (chondroid matrix mineralization) in the vertebral body of L4. (e) Sagittal *T*_1_ fat-sat after contrast, performed after 2 months. Surgical manipulation characterized by magnetic susceptibility artifacts at L3–L5 levels. There was an increase in the posterior component of the infiltrative lesion at the the topography of the vertebral body of L4, with heterogeneous contrast enhancement.

The patient was a heavy smoker, with no other comorbidities. Initial MRI demonstrated an infiltrative mass, affecting the vertebral body and pedicles of L4, with extension to the vertebral canal, with heterogeneous enhancement after contrast administration (Figure 1a,b). Tumor extended into the distal third of the inferior vena cava and the left iliopsoas muscle.

CT scan was also performed (Figure 1c). There was a lytic lesion with indistinct borders, in the vertebral body of the fourth lumbar vertebra (L4), associated with a soft tissue component that dislocated the aorta and the inferior vena cava. There was a typical “ring-and-arc” chondroid matrix mineralization in the vertebral body of L4 (Figure 1d).

The patient underwent a needle transpedicular biopsy of the vertebral lesion and a malignant infiltrative bone neoplasm was diagnosed, with chondroid differentiation and several areas of necrosis.

Three weeks after the needle biopsy, a posterior lumbar approach was performed, with installation of pedicle screws at L3 and L5 and the posterior elements were removed. The anterior large vessels were dissected from the spine and an “en bloc” resection of L4, reconstruction of the lumbar spine with a titanium cage and antero lateral plate were done.

**Figure 2.**
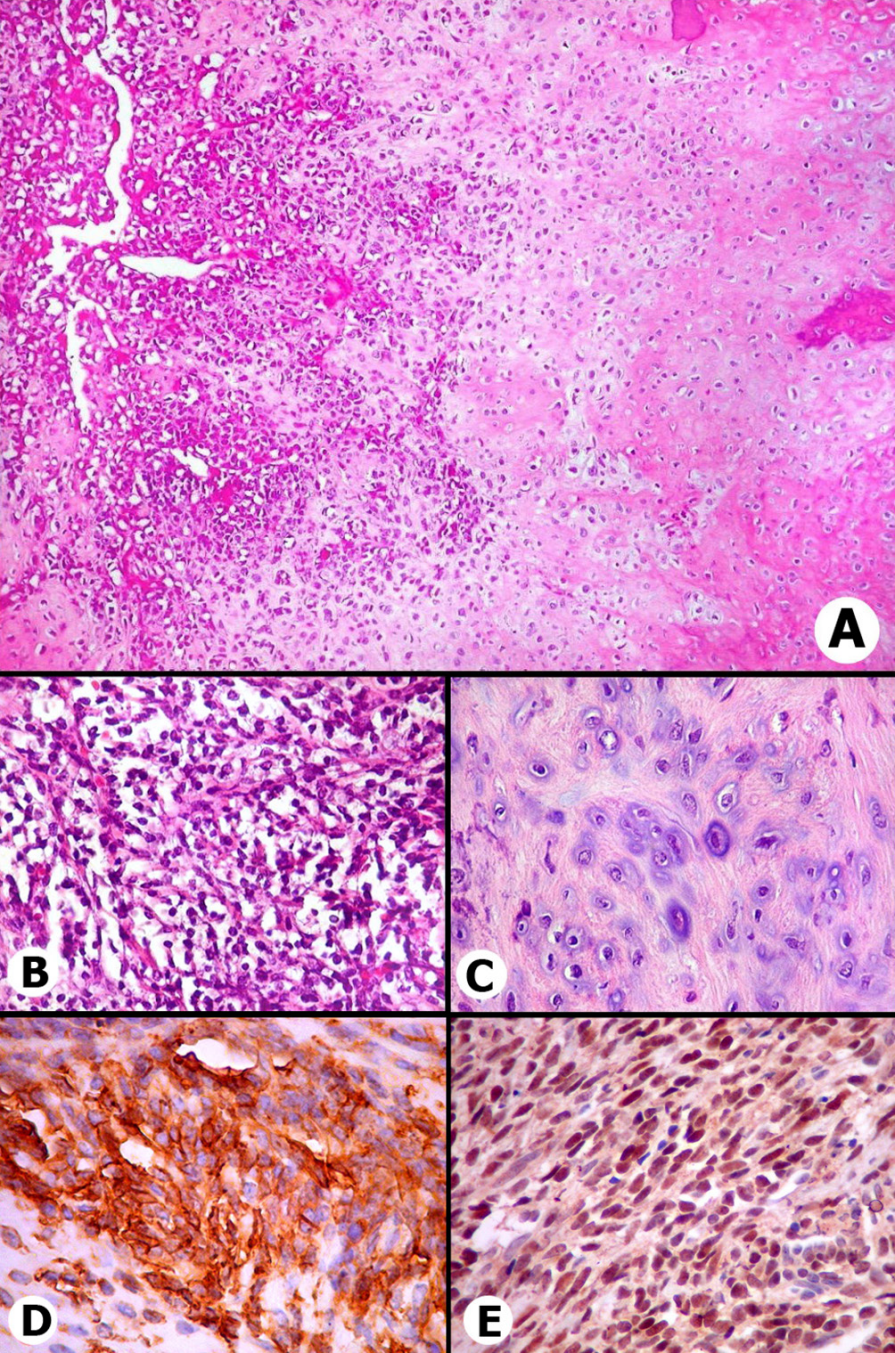
Anatomopathological images of mesenchymal chondrosarcoma in vertebral body. (A) Transition between poorly differentiated area (left) and well-differentiated tumor with cartilaginous features. At upper left, small dilated tortuous vessels with hemangiopericytomatous-like appearance. HE × 100. (B) Detail of poorly differentiated area showing small cells with dense nuclei and scanty cytoplasm in solid arrangement and virtually no interstitial matrix. HE × 400 (C) Cartilaginous differentiation. Cells resemble mature chondrocytes lodged in small cavities of the homogenous cartilaginous matrix. Nuclei are larger with slight to moderate pleomorphism. HE × 400 (D) MIC2 (C99) gene expression showing membranous positivity of tumor cells in the immature component of the neoplasm. × 400. (E) Strong nuclear expression of SOX9 protein mainly in the small cell component of mesenchymal chondrosarcoma. × 400.

Histopathological examination of the resected L4 vertebral body revealed an infiltrative malignant biphasic neoplasia composed of ample ill-defined areas of atypical cartilage with well to moderate differentiation intercepted by a variable quantity of poorly undifferentiated small cells with prominent capillary vasculature, exhibiting hemangiopericytoma-like features (Figure 2A–C). Involvement of adjacent soft tissues was noted and the surgical margins were affected. The tumor showed immunoreactivity for CD99/MIC2 protein on immature cells (Figure 2D); S-100 protein was expressed in cartilaginous areas and SOX-9 was positive in both components, but mainly in primitive mesenchymal cells (Figure 2E). No expression was found for epithelial markers (CKs; EMA) and for lymphocytic neoplastic elements. These morphological findings and immunophenotype of the tumor cells are consistent with mesenchymal chondrosarcoma (high histological grade).

**Figure 3.**
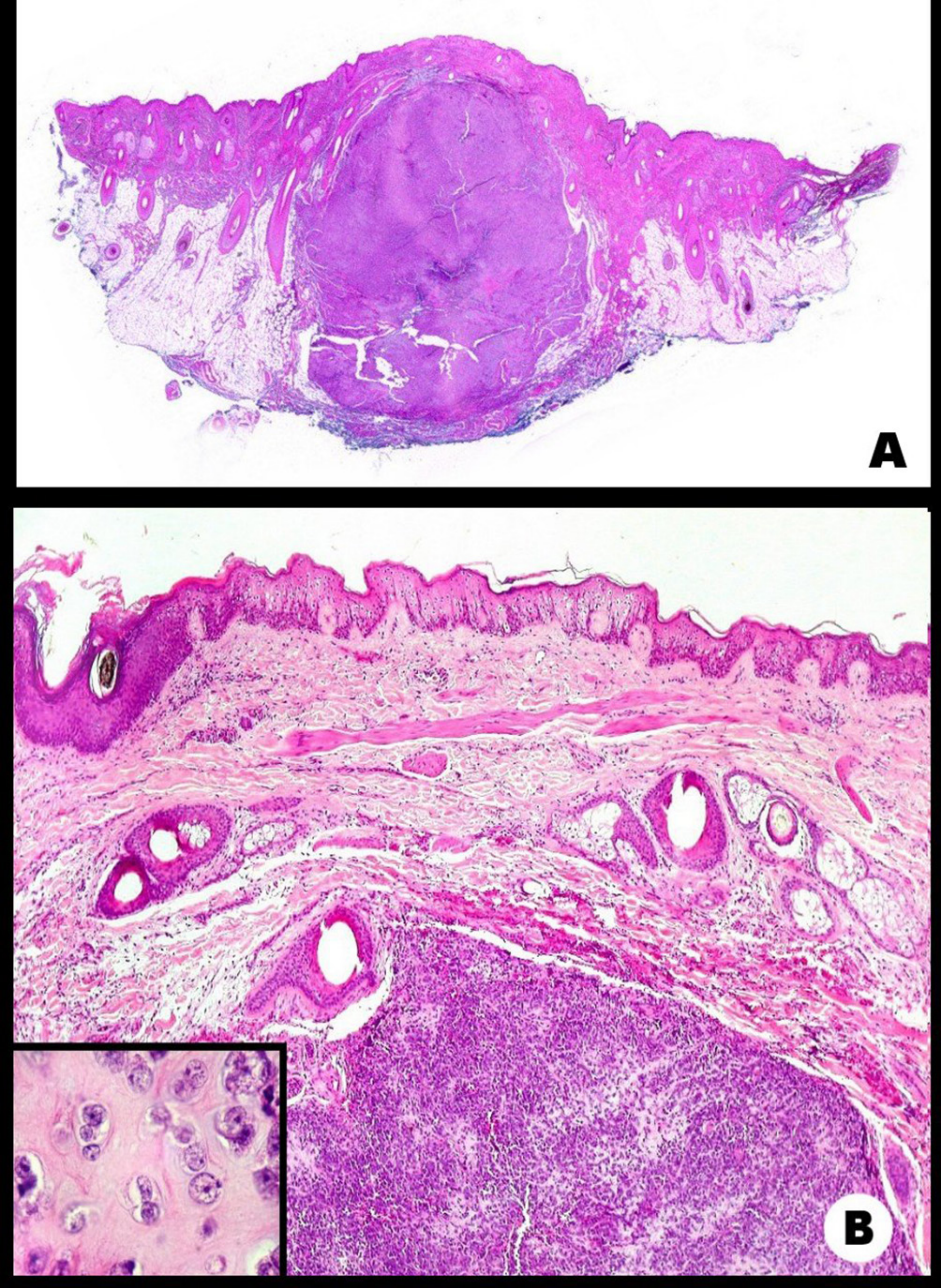
Anatomopathological images of metastasis of mesenchymal chondrosarcoma in scalp. (A) Scanned slide of well delimited tumor nodule in hypodermis, pressing on the epidermis and dermis. Epidermal adnexa and hypodermal adipose tissue visible on either side. HE × 20 9B) Metastatic tumor is predominantly immature. HE × 100; *inset*—area of cartilaginous differentiation. ×400.

Patient was discharged after 10 days of the main procedure with controlled pain and able to walk without assistance. He was seen in our outpatient facility after 20 days with moderate back pain and normal neurological examination. About 2 months after the spinal reconstruction, he developed a progressive bladder incontinence, bilateral leg weakness and severe back pain. A new MRI was performed (Figure 1e) confirming progression of the disease. Additionally, a right occipital scalp lesion was visualized and biopsied, evidencing malignant immature cell neoplasm with chondroid areas, occupying the dermis and hypodermis, compatible with cutaneous metastasis (Figure 3). He received local radiotherapy, systemic chemotherapy, and was able to walk with sticks after 2 months, with mild back pain.

After 5 months of hospital discharge, the patient underwent PET-CT, which showed progression of the disease, characterized by metastatic implants in the skin and subcutaneous (occipital and below the angle of the mandible) muscles (serratus anterior, gluteus maximus and semitendinosus), in bones (sternum, ulna and femur) and in liver. Thus, palliative treatment was indicated with radiotherapy.

## Discussion

Epidemiologically, spinal chondrosarcoma is more common in Caucasians (with no gender preference), young (mean age 33 years), with a peripheral and lumbar spine location (typically in the posterior elements and in the vertebral body).^6, 7^ Some risk factors, such as malignant transformation of chondromas and association with hereditary multiple exostoses were not observed in our patient.

This patient had a previous history of low back pain but did not undergo proper clinical radiological evaluation. New and persistent back pain of more than 3 months should be investigated preferentially with MRI.^8^

Considering radiological evaluation of vertebral body sarcomas, CT scan is the best method to detect the matrix mineralization (typical “ring-and-arc” chondroid matrix mineralization) and it can also evaluate aggressive features (such as cortical destruction and soft-tissue extension). MRI is better to evaluate the extension of the sarcomas (particularly involvement of the spinal canal) and the nature of the lesion (*T*_1_ weighted images show low to intermediate signal intensity, with enhancement that varies from homogeneous to heterogeneous, sometimes with ring and arc pattern).^4^ Currently, there are no radiographic features able to differentiate the different histological types of chondrosarcoma, such as mesenchymal, myxochondrosarcoma or a cartilage containing meningioma.^9^

 There are several other diseases that can be considered in the differential diagnosis of spine chondrosarcoma, such as angioblastic meningioma, osteosarcoma, Ewing’s sarcoma,^6^ synovial chondromatosis, chondroblastic osteosarcoma and chordoma.^10^In the case of differentiation between chondrosarcoma and chordoma, both can have abundant extracellular matrix and positivity for S-100 protein, but only the chordoma will co-express epithelial markers (*i.e.* cytokeratins, EMA) and brachyury.^11^

Microscopically, a bimorphic pattern is characteristic of mesenchymal chondrosarcoma, but when this tumor is formed by predominantly mature cartilage and minimal atypia of the chondrocytes, it can be mistaken for benign cartilage lesions, or misclassified as a well-differentiated conventional chondrosarcoma or chondroblastic osteosarcoma.^12^ A wide representation of the lesion is recommended, as well as attention to detect the undifferentiated component between larger cartilaginous areas of the tumor. Diagnosis of osteosarcoma needs confirmation of neoplastic osteoid among the cartilage.^10^ On the other side the nonchondroid part of a mesenchymal chondrosarcoma can cause confusion with Ewing’s sarcoma and related tumors, small cell osteosarcoma, as well with embryonal rhabdomyosarcoma and malignant lymphoma involving bone.^12^ None of these tumors usually presents cartilage, except as foci of metaplasia in Ewing’s Sarcoma.^12^

The immunohistochemical markers employed include S-100 protein, which is typically expressed only on cartilaginous areas of the mesenchymal chondrosarcoma. CD99/MIC2 protein is confined to small cell component but is also demonstrated in these other small round cell tumors. A valuable marker, as SOX9, shows nuclear positivity in both areas, but mainly on the immature component (chondroprogenitor cell’s phenotype) of mesenchymal chondrosarcoma, differentiating it from other small cell tumors.^12^ However, it is not totally specific, because SOX9 can be found in any neoplasm of cartilaginous lineage. The lack of FLI1 expression can be used as an additional biomarker to differentiated between mesenchymal chondrosarcoma and Ewing’s sarcoma. Also, positivity for the break-points and fusion genes involving EWSR1 is typical only for this last tumor.^12^ A promisor HEY1-NCOA2 fusion genes were identified in mesenchymal chondrosarcoma and are absent in other chondrosarcoma subtypes.^13^ Rhabdomyosarcomas and lymphomas involving bone can be excluded by the absence of their respective skeletal muscle and lymphoid biomarkers.^12^

Finally, hemangiopericytoma and other primary spindle cell malignant tumors, mainly with hemangiopericytomatous vasculature, that arise in bone, must be considered in differential diagnosis, but none show the cartilaginous components that are present in classical mesenchymal chondrosarcomas.^12^

Therefore, the present case was confirmed by anatomopathological and immunohistochemical analysis, after clinical and radiological suspicion.

Considering treatment of primary spinal tumors, the best option is total surgical resection, with vertebrectomy and spinal reconstruction, for isolated lesions. In the case of these tumors, specifically, radiotherapy and chemotherapy have no significant effect, and therefore are not part of the standard treatment.^10^ However, late diagnosis may limit a curative procedure, once local or distant dissemination will occur with disease progression, such as in the presented case.

The prognosis of spine chondrosarcoma is usually poor, although most have low grade (Grade 1 or Grade 2). As a poor prognostic factor, compromised margins can be associated to metastasis, especially to the lungs. In this case, the patient presented metastasis to an unusual site, the skin.

## Learning points

- Persistent back pain should be investigated preferentially with a MRI or CT.
- Although rare, chondrosarcomas should be suspected in young adults presenting with mass arising from the vertebral body with cortical disruption and ring and arc pattern of calcifications and foci of enhancement.
- CT and MRI are important to surgical planning in chondrosarcomas.
- MRI is better to delimitate the extension in vertebral bony tumors, particularly, the epidural component and the relation with the cauda equina roots.
